# Plasma Total Antioxidant Capacity and Cardiometabolic Risk in Non-Obese and Clinically Healthy Young Adults

**DOI:** 10.5935/abc.20170095

**Published:** 2017-08

**Authors:** Jamille Oliveira Costa, Cecília M. Passos Vásquez, Gleiciane de Jesus Santana, Natanael de Jesus Silva, Juciene de Matos Braz, Amélia M. Ribeiro de Jesus, Danielle Góes da Silva, Luana Celina Seraphim Cunha, Kiriaque Barra Ferreira Barbosa

**Affiliations:** Universidade Federal de Sergipe (UFS), Aracaju, SE - Brazil

**Keywords:** Cardiovascular Diseases, Risk Factors, Metabolic Syndrome, Oxidative Stress, Antioxidants, Young Adult

## Abstract

**Background:**

The oxidative biomarkers play an important role in the genesis of cardiometabolic risk-related processes.

**Objective:**

To investigate the total antioxidant capacity of plasma and its association with cardiometabolic risk in non-obese and clinically healthy young adults.

**Methods:**

University students of the state of Sergipe, Brazil, aged between 18 and 25 years, were recruited for this study from May of 2013 and October of 2014. Anthropometric, clinical and biochemical parameters were measured and analyzed using protocols which were previously standardized and described in the literature. The measurement of plasma total antioxidant capacity was based on the ability that all the antioxidants present in the sample (plasma) have to inhibit the oxidation of the oxidizable substrate ABTS (2,2`- Azino-di-[3-ethylbenzthiazoline sulphonate]) to ABTS•+ by metmyoglobin.

**Results:**

Approximately 25% of the sample presented more than one component of cardiometabolic risk. Low HDL-cholesterol was the most prevalent component. Compared to absence of components, the subjects with at least one component presented greater body weight and waist circumference, higher levels of diastolic blood pressure and fasting glucose, greater total cholesterol/HDL-c ratio, and lower levels of HDL-c (p < 0.05). Fasting glycemia was the only parameter which was associated with total antioxidant capacity (R^2^ = 0.10; β = 0.17; p = 0.001).

**Conclusions:**

The plasma total antioxidant capacity was not able to predict the cardiometabolic risk components due possibly to the establishment of compensatory mechanisms that become activated in physiological conditions.

## Introduction

The presence of cardiometabolic risk (CMR) components, such as systemic arterial hypertension, hyperglycemia, abdominal obesity, and lipid profile alterations (e.g. high triglycerides and low HDL-c)^1,2^ has been strongly associated with oxidative stress (OS) which is established by the increased expression of oxidizing substances to the detriment of antioxidants.^3^

The association between OS and the CMR components has been evidenced due to intense production of reactive oxygen species (ROS) from metabolic alterations, such as increased insulin resistance and visceral adiposity.^4^

Total antioxidant capacity (TAC) of plasma is an important biomarker of OS since it defines the synergistic effect between the various antioxidant compounds in the sample.^5^ The presence of CMR components and chronic non-communicable diseases (NCD) has been associated to lower TAC levels in the population.^6^ Studies have demonstrated that this decrease in the TAC occurs because of the greater amount ROS as compared to the antioxidant compounds.^7,8^

The presence of CMR components in individuals with NCD is predictable and largely described in the literature since these components are predisposing factors of NCDs.^9^ OS and inflammation have also been associated with CMR and NCD.^4^ However, there is still a dearth of investigations with representative data about frequency of CMR components and their predisposing factors among healthy populations in Brazil.

Therefore, the association between TAC and anthropometric, clinical, and biochemical components of the CMR in non-obese, clinically healthy young adults is still not well elucidated in the literature. The hypothesis of this study is that decreased TAC increases CMR, even in non-obese, healthy young adults. Evaluating changes in CMR components, as well as in their predisposing factors, may be a preventive measure to the development of NCDs, since clinical consequences of NCDs, such as clinical complications, increasing prevalence of premature death, and its social and economic costs may be prevented by interventions.^10^ The objective of the study was to evaluate the association between TAC of plasma and CMR components in non-obese and clinically healthy young adults.

## Methods

### Study design

This is a cross-sectional study on a convenience sample. The volunteers’ recruitment occurred through invitations by email, posters, and classroom visits. The data collection was performed between May of 2013 and October of 2014 at two universities, one public and one private, located in the city of Aracaju, Northeast, Brazil.

### Participants

Non-obese, clinically healthy young adults, who were students of schools of health sciences, aged between 18 and 25 years of both sexes, participated in the study. Exclusion criteria included the evidence of any disease related to OS, chronic inflammation, gestation, lactation, water-electrolyte imbalance, and self-reported changes in body composition or in absorption and/or metabolism of nutrients. Exclusion criteria still included recent use of medicaments and/or dietary supplements, follow-up of nutritional treatment that may affect energy balance, food consumption, lipid profile, plasma insulin levels, and metabolism of glucose; regular use of birth control pills in the 2 months before the participation in the study; unstable body weight in the past 6 months (10% variation above or below allowed); being an elite athlete or planning to change lifestyle during the period of the study; and follow-up of special diets (e.g. vegetarian diet, Atkins diet, etc.) in the 3 months prior to the participation of the study.

The sample was calculated according to Miot (2011),^11^ considering a prevalence of 9.9% for high waist circumference among university students,^12^ significance level of 5.0%, sampling error of 5.0% and population size of 8,951, considering the number of university students enrolled in health majors. A minimum sample size of 135 individuals was estimated.

### Anthropometric and body composition parameters

Height was measured to the nearest 1 mm using a stadiometer (Altura Exata, Minas Gerais, Brazil). Weight was measured to the nearest 100 grams using an electronic digital balance (Líder, P 180M, São Paulo, Brazil) with maximum capacity of 180 kilograms. Body Mass Index (BMI) was calculated by dividing body weight (kg) by height squared (m) and classified according to the cutoff points proposed by the World Health Organization (WHO).^13^

Waist circumference was measured to the nearest 1 mm between the last rib and the iliac crest using a flexible and inelastic tape measure.^13^

Triceps, biceps, subscapular, and suprailiac skinfolds thickness were measured to the nearest 1 mm using a skinfold caliper (*Lange caliper, Cambridge Scientific Industries Inc., Cambridge, Maryland, USA*). Truncal fat percentage was calculated from the ratio of the sum of subscapular and suprailiac skinfolds and the sum of the four skinfolds.^14^

Body fat percentage was obtained by bioelectrical impedance analysis using a quadrupole device (Biodynamics model 310, Washington, USA), from which body fat and fat-free mass were calculated in kilograms. 

### Biochemical measures

Blood collection was performed by venipuncture after fasting of 12 hours and no intake of alcohol, coffee or tea for 24 hours. Samples of heparin and plasma were separated by centrifugation at 2,465 g to 5°C for 15 minutes and stored at -80°C.

Serum concentration (mg/dL) of glucose, total cholesterol, high-density lipoprotein (HDL-c), and triglycerides (TG) were analyzed by a colorimetric or turbidimetric assay by an automatic analyzer using specific assay kits.

TAC in plasma was determined by colorimetric assay using a specific assay kit (Cayman Chemical, Ann Arbor, MI, catalog no. 709001). The assay was based on the ability that all the antioxidants present in the sample (plasma) have to inhibit the oxidation of the oxidizable substrate ABTS (2,2`- Azino-di-[3-ethylbenzthiazoline sulphonate]) to ABTS•+ by metmyoglobin. The amount of oxidized substrate (ABTS•+) was monitored by absorbance reading at 750 nm. The decrease in absorbance at 750 nm was directly proportional to the concentration of antioxidants in plasma expressed as mM Trolox equivalents, a synthetic water-soluble analogue of vitamin E.

### Blood pressure

Systolic and diastolic blood pressure levels were measured to the nearest 2 mmHg using a mercury sphygmomanometer according to Perloff et al.^15^

### CMR components

The CMR components were diagnosed according to the criteria of the International Diabetes Federation (2005):^1^ abdominal obesity (waist circumference >80 cm for women and > 94 cm for men); fasting hyperglycemia (> 100 mg/dL); hypertriglyceridemia (> 150 mg/dL); low HDL-C (< 50 mg/dL for women and < 40 mg/dL for men); and hypertension (systolic pressure > 130 mmHg; diastolic pressure > 85 mmHg).

### Food Consumption and lifestyle variables

Usual dietary intake was obtained by the application of a semiquantitative food frequency questionnaire (FFQ), developed for this study population. The Virtual Nutri software was used to quantify energy and nutrient intake. Inadequate intake was evaluated according to the recommendation proposed by the Dietary Reference Intakes (DRI) (National Research Council, NRC, 2011),^16,17^ using the Estimated Average Requirement (EAR) and the Adequate Intake (AI) values as cutoff points. Inadequate energy intake was determined by the intake < 90% or > 110% from the Estimated Energy Requirement (EER), calculated by predictive equations proposed by the Institute of Medicine (2005).^15^ Some methodological precautions were adopted around the food consumption assessment, such as the use of visual aids to assist the participants estimate the portion sizes during the FFQ application, training of interviewers, pilot test to clarify questions and inadequacies in the FFQ, and standardization of recipes.

We collected information on vitamin supplements, smoking, number of cigarettes per day, regular physical activity, and its intensity. To characterize and quantify physical activity, we used the short version of the International Physical Activity Questionnaire (IPAQ), which is recommended by the World Health Organization and has been validated in Brazil by the Center of Studies of the Physical Fitness Research Laboratory of São Caetano do Sul - CELAFISCS.^18^

### Statistical analysis

Continuous variables were presented as mean ± standard deviation while categorical variables as absolute (n) or relative frequency (%).

Kolmogorov-Smirnov test was used to verify the normality of the distribution. Unpaired Student’s t-test was adopted to compare the categorized groups by the presence of the CMR components. To track the correlation between TAC and other variables of interest related to the components of the CMR, we used Pearson test. Multivariate linear regression was performed with the fasting glucose values (mg/dL) as being the dependent variable and TAC value (mM), sex and age as being the independent variables. A 95% confidence interval was used to describe the values of the linear regression coefficient (β).

Statistical significance was accepted at p < 0.05. All analyses were conducted using Statistical Package for Social Science, SPSS version 20.0, for Windows.

### Ethical aspects

The study was approved by the Human Research Ethics Committee of the Federal University of Sergipe (C.A.A.E.: 0113.0.107.000-11).

In accordance with the principles of the declaration of Helsinki, all volunteers were informed about the study protocol and then signed the consent form. The volunteers were informed about the methods and procedures used in the data collection, the possible benefits and inconveniences, the privacy of results and the voluntariness of participation.

## Results

A total of 139 non-obese and clinically healthy young individuals, aged 21.4 ± 1.9 years, participated of the study. Women predominated in the distribution of gender (77%). The anthropometric, clinical and biochemical parameters are described in Table 1.

**Table 1:** Demographic, anthropometric, clinical and biochemical characteristics (mean and standard deviation) of non-obese and clinically healthy young adults

Total (n=139)	X	SD
Age (years)	21.4	1.9
Weight (kg)^a^	55.9	7.4
BMI (kg/m^2^)^a^	20.6	2.1
WC (cm)^a^	71.1	5.6
TSF (mm)^a^	18.6	6.8
BSF (mm)^a^	9.6	5.4
SISF (mm)^a^	16.1	6.5
SSF (mm)^a^	14.8	4.4
Total fat (%)^a^	23.0	9.7
Truncal fat (%)^a^	53.2	7.0
Fat mass (kg)^a^	12.7	5.5
Fat-free mass (kg)^a^	43.1	8.6
SBP (mmHg)^b^	108.8	8.0
DBP (mmHg)^b^	74.8	7.8
Fasting glucose (mg/dL)^c^	85.6	8.4
Total cholesterol (mg/dL)^d^	170.7	38.5
HDL-c (mg/dL)^d^	56.0	10.9
LDL-c (mg/dL)^d^	98.9	33.0
Triglyceride (mg/dL)^d^	78.4	35.2
Total cholesterol/HDL-c^d^	3.0	0.7

BMI: body mass index; WC: waist circumference; TSF: triceps skinfold; BSF: biceps skinfold; SISF: suprailiac skinfold; SSF: subscapular skinfold; SBP: systolic blood pressure; DBP: diastolic blood pressure; HDL-c: high-density lipoprotein; LDL-c: low-density lipoprotein.

a n = 123;

b n = 122;

c n = 136;

d n = 137.

Reference values: waist circumference < 80 cm for women and < 94 cm for men; fasting glucose ≤ 100 mg/dL; triglyceride ≤ 150 mg/dL; HDL-c > 50 mg/dL for women and > 40 mg/dL for men; SBP ≤ 130 mmHg; DBP ≤ 85 mmHg.

Although the individuals of this study were non-obese and clinically healthy, they already presented CMR components. About 15% (n = 20) of the sample had low HDL-c concentrations followed by high diastolic blood pressure levels (n = 9; 7%), triglyceride (n = 8; 6%), glucose (n = 6; 4%), and abdominal obesity (n = 3; 2%). Almost one-quarter of the sample (n = 34; 24.5%) had at least one component of the CMR followed by 2 (n = 11; 8%) and 3 or more CMR components (n = 1; 0.7%), respectively.

Among the nutrient intake inadequacies were considerable those relating to the consumption of saturated fat (n = 114; 92%), fiber (n = 89; 72%), and vitamin D (n = 107; 86%).

The individuals were categorized by the presence of the CMR components (Table 2). Those with at least one component showed greater body weight, TG/HDL-c ratio, and total cholesterol/HDL-c ratio when compared with the individuals who did not present any of the CMR components (p < 0.05).

**Table 2:** Demographic, anthropometric, clinical and biochemical characteristics (mean and standard deviation) according to the presence of cardiometabolic risk components among non-obese, clinically healthy young adults (n = 139)

	No component		≥ 1 component		
	X	SD	X	SD	p
Age (years)	21.4	2.0	21.9	1.8	0.73
Weight (kg)^a^	54.5	6.7	60.6	7.7	< 0.01
BMI (kg/m^2^)^a^	20.5	2.0	29.0	2.3	0.28
Total cholesterol (mg/dL)^d^	171.9	39.5	166.8	35.5	0.50
LDL-c (mg/dL)^d^	99.1	34.9	98.3	26.7	0.90
Triglyceride/HDL-c (mg/dL)^d^	1.2	0.5	2.0	0.9	0.00
Total cholesterol/HDL-cd	2.9	0.6	3.5	0.8	< 0.01
TAC (mM)^d^	3.1	0.6	2.9	0.8	0.23

BMI: body mass index; TAC: total antioxidant capacity; LDL-c: low-density lipoprotein. Data present as mean ± standard deviation (X ± SD); statistical signicance level of 5%; Student's t-test.

a n = 123;

b n = 122;

c n = 136;

d n = 137

**Figure 1:**
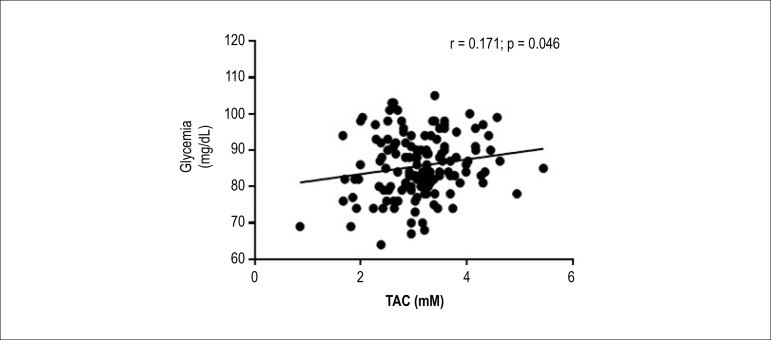
Pearson correlation between plasma total antioxidant capacity (TAC) and fasting glycemia values (n = 139).

Glycemia was the only CMR component which correlated with plasma TAC (Figure 1).

After adjusting the multivariate linear analysis by sex and age, fasting glycemia was positively associated with the TAC values (R^2^= 0.10; β = 0.17; p = 0.001). TAC had a positive predictive effect on the serum glucose levels. An increase by 1 unit in the levels of TAC was associated with an increase by 0.17 mg/dL in fasting glycemia. It is noteworthy that 10.0% of the increase in glycemia was due to the effect of TAC (Table 3).

**Table 3:** Multivariate linear regression analysis with the glycemia (mg/dL) as dependent variable adjusted by sex and age (n = 139)

	β (95% confidence interval)	p
TAC (mM)	0.174 (0.135-3.957)	0.030
Sex	0.267 (2.099-8.553)	0.001
Age	–0.132 (-1.243-0.128)	0.110

R^2^ = 0.100, p = 0.001. TAC: total antioxidant capacity.

## Discussion

The study about the CMR components has attracted attention since they are strongly related to the development of diseases associated with insulin resistance and cardiovascular events, which are the major causes of morbidity and mortality in the population.^19^ These diseases have direct and indirect impact on social and state economy, including low productivity, loss of working days, damage to the productive sector, and intangible costs of people affected by these conditions.^10,20^

Determining the presence of CMR components in young populations is relevant for the early diagnosis, and establishment of specific interventions and preventive measures. In our study population, although the prevalence of alterations in the outcome measures was low - according to the proposed reference values - almost one-quarter of the sample had at least one component of the CMR. Low HDL-c was the most prevalent component (15%). On the other hand, studies on college students, both in Brazil^20^ and in other countries,^21,^ ^22^ have found higher prevalence. This controversy may be related to the characteristics of our population, composed of physically active (65%), non-smoking, university students from health sciences area, mainly nutrition (39%), and with low prevalence of alcohol consumption.

The prevalence of CMR components in college populations in national^23^ and international^21,22^ studies varied from 30 to 77% among individuals with at least one risk component, from 12 to 13% for those with two, and from 3 to 16% for those with three components. Low HDL-c and high blood pressure are the most prevalent CMR components.

The early development of these components in young adults has been attributed to poor eating habits, commonly observed in young populations, due to factors related to this life stage, including independence, inability to make healthy food choices, lack of time, convenience, costs, and influence of both physical and social environments.^24^ The result of this combination is the greater weight gain in the first year of college when compared to adulthood.^25^

In this study, it was observed that the presence of at least one risk component in the study population was associated with greater anthropometric (body weight) and biochemical (TG/HDL-c and total cholesterol/HDL-c ratios) parameters. These results are corroborated by studies conducted with college students from Brazil^23^ and from other countries.^21,22^

TG/HDL-c ratio has been largely discussed as an atherogenic risk indicator for predicting the development of coronary artery disease,^26^ acute myocardial infarction,^27^ and atherosclerosis.^28^ Studies have showed that high values of TG/HDL-c are correlated with increased CMR in the population.^22,29^ In addition, TG/HDL-c ratio has been positively correlated with biochemical (total cholesterol, LDL-c, and TG) and anthropometric parameters (BMI, waist circumference, and body fat percentage) and negatively with HDL-c.^30^

The presence of the CMR components, such as abdominal obesity,^31^ hypertriglyceridemia, low HDL-c and hypertension,^32,33^ has been associated to the development of NCDs, especially, type 2 diabetes, cardiovascular diseases, and cancer. In Brazil, a study was conducted to evaluate the association between cardiovascular risk factors and anthropometric indicators in patients with NCD.^34^ The authors found that 74% of the sample presented glycemia > 100 mg/dL, 56% low HDL-c, 82% high waist circumference, and 78% overweight.^34^

Excessive ROS production is another factor that has been associated with the presence of CMR components and, consequently, to the development of NCDs.^4,6^ Several studies have identified the association between plasma TAC -an important biomarker of OS for expressing the synergistic action between various antioxidant compounds^5^ - and alterations in the anthropometric, clinical and biochemical CMR components.^6^ It is worth mentioning that all these studies were conducted with individuals with NCDs. There is still a dearth of studies with healthy individuals.

Although the individuals of the present study were non-obese and clinically healthy, it was possible to identify an association between TAC and fasting glycemia after adjustment by sex and age (R^2^= 0.10, β = 0.17, p = 0.001). The positive correlation between TAC and fasting glucose found in the study does not corroborate with the literature.^35^ Hyperglycemia increases the expression of OS by the increase of NADPH concentrations and ROS production due to the intense mitochondrial metabolism of glucose.^36^ This results in an increased production of electron donors (FADH2 and NADH) in the Krebs cycle and, hence, in a high mitochondrial membrane potential (DmH+) by pumping protons across the inner membrane, inhibiting electron transport at complex III, and increasing the half-life of free-radical intermediates of coenzyme Q (ubiquinone) which reduces O_2_ to superoxide. Thus, studies have shown a negative correlation between fasting glycemia and plasma and dietary TAC,^37^ as well as greater amount of products from oxidative reactions, which reduce the level of the substances that make up the antioxidant system.^38^ However, all these studies were conducted with individuals with NCD already established.

Due to the characteristics of the participants of this study -young, clinically healthy, and non-obese, and the high TAC, one may suggest the establishment of an adaptive mechanism based on the evidence that the increase of 1 unit in the TAC levels (1mM) is associated to the increase of 0.17 mg/dL in the fasting glycemia levels, *i.e*., increased glycemia in homeostasis would determine a compensatory increase of the TAC. This occurs through negative feedback which may activate the enzymatic pathways of the antioxidant system to reduce the intracellular levels of ROS, thereby minimizing oxidative damage.^39^ The findings by Demirbag et al.^35^ corroborate this assumption. The increase in TAC becomes impracticable in pathologic conditions already set in, different from what occurs in health individuals.

The lack of associations between TAC and the other anthropometric, clinical and biochemical variables in the study may be explained by the low prevalence of alterations on these parameters and by the characteristics of the studied population: young, predominantly women, non-obese, clinically healthy, physically active, students of health sciences, low alcohol consumption, non-smokers, and markers of adiposity - waist circumference (71.1 ± 5.6 cm) and body fat percentage (23.0 ± 9.7%) - below the risk for triggering metabolic alterations. Nevertheless, it is worth pointing out the high TAC value found in the present study (3.10 ± 0.71; median = 3.09 mM) compared to the values found by Barbosa et al.^40^ in young adults (1.60 mM). This result may be associated with the low prevalence of behavioral risk factors, such as sedentary life style (36%), low consumption of alcohol, being non-smoker, in addition to be university students in health sciences.

Some limitations in this study must be acknowledged: the sample loss of some variables due to incomplete information and/or study dropout; the assessment of food consumption by instruments available in the literature are subject to error because of their large inter- and intra-individual variability, as well as the dependence on the respondents' memory about past habits, low accuracy in quantifying the intake due to the use of standardized measures and food lists. Finally, the methods used to evaluate plasma TAC strictly reflect chemical reactions *in vitro*, without similarity to biological systems. Their results should therefore be interpreted with caution since they do not measure bioavailability, *in vivo* stability, retention of antioxidants in the tissues, and *in situ* reactivity.

## Conclusions

In this study, CMR components were present in some young, clinically healthy, non-obese, and high-TAC adults.

The observed positive correlation between plasma TAC and fasting glycemia suggests the establishment of an adaptive mechanism. The increase in glycemia in a biological system, in homeostasis, would determine a compensatory increase of the plasma TAC.

Thus, different from what occurs in populations with NCD already set in, TAC was not associated with CMR components in this sample of young, non-obese and clinically healthy individuals due possibly to the establishment of compensatory mechanisms that become activated in physiological conditions.
